# Within-Person Fluctuations in Momentary Loneliness Among People With Schizophrenia

**DOI:** 10.1177/00207640251383127

**Published:** 2025-10-31

**Authors:** Miya M. Gentry, Daniel Zoleikhaeian, Henry J. Wong, Molly A. Patapoff, Allison P. Williams, Sophia Ross, Xin M. Tu, Barton W. Palmer

**Affiliations:** 1 San Diego State University/University of California San Diego Joint Doctoral Program in Clinical Psychology, San Diego, CA, USA; 2 Department of Psychiatry, University of California San Diego, La Jolla, USA; 3 Sam and Rose Stein Institute for Research on Aging, University of California, San Diego, La Jolla, CA, USA; 4 University of California San Diego Master of Science Program in Biostatistics, San Diego, USA; 5 Herbert Wertheim School of Public Health & Human Longevity Science, University of California, La Jolla, CA, USA; 6 Veterans Affairs San Diego Healthcare System, VISN 22 Mental Illness Research, Education, and Clinical Center (MIRECC), San Diego, CA, USA

**Keywords:** schizophrenia, loneliness, ecological momentary assessment

## Abstract

**Background:**

Loneliness is highly prevalent among individuals with schizophrenia and contributes to poor functional and clinical outcomes. However, most research to date has relied on trait-based assessments, providing limited insight into the dynamic nature of loneliness experiences.

**Aims:**

The current study employed ecological momentary assessment (EMA) to examine momentary loneliness and its variability in people with schizophrenia relative to a comparison group of participants without a history of serious mental illness (NC).

**Methods:**

Participants included 104 adults (39 with schizophrenia or schizoaffective disorder and 65 NC). Participants completed up to 28 EMA surveys over seven consecutive days.

**Results:**

Participants with schizophrenia reported significantly higher trait and momentary loneliness, as well as greater between-person and within-person variability in momentary loneliness. Trait loneliness was moderately associated with momentary loneliness but did not account for the elevated within-person variability observed in the schizophrenia group.

**Conclusions:**

Findings underscore the importance of considering both chronic and dynamic features of loneliness in schizophrenia and highlight the potential value of real-time assessment for informing targeted interventions.

## Introduction

Recent years have brought substantial attention to the “epidemic of loneliness” in the general population (Murthy, 2023), and more recently, there has been growing empirical attention to the impact of loneliness on schizophrenia (Patterson et al., 2024; Randolph et al., 2025). People with schizophrenia report significantly higher rates of loneliness than the general population, with some estimates as high as 80% (Badcock et al., 2015; Stain et al., 2012). However, defining and measuring chronic loneliness remains a challenge, as there is no consensus on a diagnostic threshold for problematic loneliness (Hussain & Palmer, 2024). Most of the existing literature on schizophrenia and the general population has relied on trait-based measures of loneliness, such as the UCLA Loneliness Scale (UCLA-LS) (Russell, 1996), which assesses enduring perceptions of social disconnection rather than capturing momentary shifts in loneliness experiences. With some notable exceptions (Culbreth et al., 2021; Moran et al., 2024; Mote & Fulford, 2020), much less is known about state loneliness and its within-person fluctuations, particularly in schizophrenia.

Prior research in non-serious mental illness (SMI) populations has shown that state (momentary) loneliness fluctuates in response to social context, cognitive processes, and affective states (Maes & Vanhalst, 2024; McComb et al., 2020). Although acute loneliness appears to be an adaptive response to social isolation (Cacioppo & Cacioppo, 2018; Maes & Vanhalst, 2024), studies have found that momentary fluctuations in loneliness are associated with depression, anxiety, and perceived stress, even when controlling for trait loneliness (Buecker et al., 2024; Moran et al., 2024). These findings suggest that loneliness variability itself, rather than just mean levels of loneliness, may be a clinically relevant construct. However, the degree of intra-individual variance in state loneliness among people with schizophrenia remains largely unexplored.

To date, only a few studies have examined momentary loneliness in people with schizophrenia (Culbreth et al., 2021; Moran et al., 2024; Mote & Fulford, 2020). These studies used ecological momentary assessment (EMA), a method that captures real-time fluctuations in social and affective experiences. A few prior studies employing EMA surveys have found that people with psychosis report higher overall levels of loneliness compared to non-SMI comparison groups on EMA surveys (Culbreth et al., 2021; Moran et al., 2024). However, less is known about the extent to which loneliness fluctuates within individuals over time. Understanding whether loneliness variability is heightened in schizophrenia could provide insight into the everyday dynamics of loneliness in this population, which could have implications for managing loneliness in ways that yield adaptive rather than maladaptive responses.

Several features of schizophrenia may help explain why loneliness could be more variable in this population. Heightened sensitivity to social interactions, inconsistent social motivation, and difficulties interpreting social cues (Cavieres et al., 2023; van’t Wout et al., 2009; Weittenhiller et al., 2021) may contribute to labile experiences of connection and disconnection in daily life, particularly during ambiguous or challenging social encounters. In turn, this volatility in loneliness may signal vulnerability to mood instability and impaired social functioning (Culbreth et al., 2021; Moran et al., 2024), or even increased risk for suicidality (Chen et al., 2023). Notably, people with schizophrenia often show elevated risk in these domains. If supported by empirical data, such variability could represent a clinically relevant target for interventions focused on emotion regulation or social connection. Conversely, if variability is not elevated, this may suggest that trait-like loneliness is the more salient clinical feature. This hypothesis-driven approach reflects a broader shift within psychosis research toward using EMA to capture dynamic fluctuations in symptoms and functioning (Cavieres et al., 2023; Moran et al., 2024; Mote & Fulford, 2020; Myin-Germeys et al., 2001; Wright et al., 2021). EMA methods often reveal distinct but complementary aspects of experience compared to traditional assessments, underscoring the need to study both stable and fluctuating features of psychopathology (Culbreth et al., 2021; Moran et al., 2024). Thus, applying this framework to loneliness may clarify under what conditions, and for whom, loneliness is most clinically relevant in schizophrenia.

The current study examined differences in loneliness variability between people with schizophrenia and a non-SMI comparison group (NC). Based on the considerations reviewed above, we hypothesized that people with schizophrenia have greater within-person variability in momentary loneliness compared to the NC participants. Additionally, as an exploratory aim, we examined whether higher trait loneliness is associated with greater momentary loneliness variability, providing insights into how stable loneliness perceptions relate to real-time loneliness experiences.

## Methods

### Participants

Data for the current study were collected as part of an ongoing study of loneliness and aging in schizophrenia. Participants for the current analyses included 104 adults, including 39 participants with schizophrenia or schizoaffective disorder and 65 participants reporting no history of SMI (NC group). People with schizophrenia were recruited through San Diego county Board-and-Care facilities, clubhouse programs in the greater county area, as well as a registry of participants in prior studies who consented to be contacted for future research participation. Recruitment for NC participants was done through community flyers and ResearchMatch.com, as well as the above-described registry of participants. The study was approved by the UC San Diego Office of IRB Administration and conducted in accordance with the ethical principles of the Declaration of Helsinki.

Due to the design of the parent study, participant enrollment was restricted to persons between the ages of 41 and 70 years. Inclusion in the schizophrenia group required having a current diagnosis of schizophrenia or schizoaffective disorder, confirmed and documented by their treating mental health provider in available medical records. Inclusion in the NC group required having no current or past history of psychosis, bipolar disorder, or major depressive disorder of sufficient severity to require hospitalization. NC status was verified through a screening interview conducted by trained research staff under the supervision of a licensed clinical psychologist (i.e., senior author). The screening included standardized questions regarding prior mental health diagnoses or treatment, as well as targeted inquiries about DSM-5 symptoms of psychosis, mania/hypomania, and major depressive episodes (American Psychiatric Association, 2013). For both groups, additional inclusion criteria included fluency in English and the ability to provide written and verbal informed consent, as documented via an IRB-approved consent process. Exclusion criteria included plans to relocate outside the county within 12 months, the presence of dementia or other neurological conditions affecting cognition, active substance use disorders, or medical conditions likely to interfere with participation in study procedures or assessments.

In addition to the above inclusion/exclusion for the parent study, inclusion criteria for the present analyses required completion of at least 25% of EMA surveys (Myin-Germeys et al., 2001) and completion of the UCLA Loneliness Scale (UCLA-LS) (Russell, 1996), Of the 162 participants enrolled in the parent study at the time of these analyses, 109 participants (66 NC, 43 schizophrenia) met the EMA completion threshold. Common reasons for insufficient EMA data included broken phones, lack of service, or persistent difficulty navigating the EMA interface. Of those 109 participants, five were missing UCLA-LS data (1 NC, 4 schizophrenia), resulting in a final analytic sample of 104 participants (65 NC, 39 schizophrenia).

### Procedures

The analyses for the current study are based on baseline data collected via EMA, as well as interview-rating-based scales and self-administered questionnaires. The EMA surveys were delivered four times per day for seven consecutive days by a web-based service (NeuroUx; Paolillo et al., 2024), resulting in a potential total of 28 surveys. For the EMA component, participants were given the option of using their smartphone (i.e., iPhone or Android) or using a study-provided Samsung Galaxy S8 Android smartphone. The surveys were delivered by a web-based program. All participants were provided with a training session at the end of their in-lab baseline visit and a training manual on operating the study-provided smartphone, if borrowed, and on completing the EMA tasks. During the 7-day period, research staff conducted as-needed check-ins to maintain adherence and to resolve participant concerns. Participants received $1.00 for each completed survey for a maximum of $28 (in addition to $105 for in-lab testing).

### Measures

*Sociodemographic Characteristics*: Information on age, gender, and race/ethnicity characteristics was gathered through self-report during interviews and/or self-administered rating scales.

*Severity of Psychopathology*: Psychotic symptom severity was assessed using the Scale for the Assessment of Positive Symptoms (SAPS; Andreasen, 1984) and the Scale for the Assessment of Negative Symptoms (SANS; Andreasen, 1989). The SAPS evaluates the presence and severity of positive symptoms such as hallucinations, delusions, and disorganized thinking. The SANS assesses negative symptoms, including affective flattening, alogia, avolition, anhedonia, and attentional impairment. Total scores were computed for each scale, with higher scores indicating greater symptom severity.

*State Loneliness*: Real-time fluctuations in loneliness were measured using a single loneliness question from a 15-item EMA-based survey, which was administered four times per day via smartphone over seven consecutive days, totaling 28 surveys. The first EMA survey was administered within 1 week of completing the UCLA-LS. Surveys came in 3-hr windows between morning, midday, afternoon, and evening, and participants were able to request a specific time of survey receipt. For the loneliness item, participants were prompted with the question, “Right now, I feel lonely. . .” rated on a five-point scale (ranging from 1 = “not at all,” 3 = “somewhat,” 5 = “very much”). The wording of this item was adapted from prior EMA studies (Goldstein et al., 2018; Manasse et al., 2018).

*Trait Loneliness*: Loneliness was measured using the 20-item UCLA Loneliness Scale—Third Edition (UCLA-LS)(Russell, 1996). The UCLA-LS is the most widely used and best-validated measure of this construct, and previous research from our colleagues indicates equivalent factor structure in people with schizophrenia compared to a non-SMI comparison group (Eglit et al., 2018). Example items include “I feel isolated from others” and “There is no one I can turn to,” which participants rate on a 4-point Likert scale ranging from “Never” to “Always.” Total scores on the UCLA-LS have a potential range of 20 to 80 (higher scores indicating worse loneliness). The UCLA-LS has excellent internal consistency (Cronbach’s *a* > .88) and test-retest reliability (r = .73) (Russell, 1996).

### Statistical Analyses

Descriptive statistics were computed for demographic variables and study measures, with independent t-tests and chi-square tests used to compare diagnostic groups on continuous and categorical variables, respectively. In the schizophrenia group, we also examined the relationship between positive and negative symptom severity and trait loneliness (i.e., UCLA-LS scores). Pearson correlations were conducted to assess whether either symptom domain was associated with trait loneliness. Although findings are somewhat mixed, prior research suggests that loneliness may vary based on age, gender, and race/ethnicity (Dahlberg et al., 2018; Taylor et al., 2025). Therefore, these variables were included as covariates in all models.

We used four different random-intercept linear mixed effects models to examine how between-subject and within-subject variances differed across the schizophrenia and NC groups. In these linear mixed effects models, the response variable EMA loneliness score was modeled by diagnostic group, age, race/ethnicity, and gender. The error after fitting these fixed effects was captured using subject-specific random intercepts and within-subject residuals. Variances of subject-specific random intercepts were used to assess the between-subject variability, while the variances of the within-subject residuals were used to assess the within-subject variability (Hedeker & Mermelstein, 2007). Each mixed effects model used one of four different variance structures, which were: (1) homogeneous between-subject and within-subject variances; (2) heterogeneous between-subject variances; (3) heterogeneous within-person variances; and (4) heterogeneous between- and within-subject variances. In this context, a homogeneous variance structure assumes that variability is equal across the schizophrenia and NC groups, while a heterogeneous variance structure allows for variability to differ between groups. After fitting the four models using restricted maximum likelihood, we used likelihood ratio tests to determine which variance structure best represented the between-subject and within-subject EMA loneliness variability across the schizophrenia and NC groups. Next, a second set of linear mixed effects models was fit to investigate the effect of adding UCLA-LS as a predictor, and the above procedure was repeated.

Finally, we calculated intraclass correlations (ICCs) to characterize the proportion of error variability due to between-subject variance versus the total (sum of between- and within-subject) variance, that is, an ICC of 1 would suggest all error variability is due to between-subject variance, while an ICC of 0 would suggest all error variability is due to within-subject variance. Inference for ICC was based on bootstrap resampling methods (Efron & Tibshirani, 1994). Welch’s T-test accounting for unequal variances was used to determine whether there was a significant difference in the ICC results between the schizophrenia and NC groups over the 1,000 bootstrapped samples.

All statistical analyses were conducted in R version 4.3.0 (R Core Team, 2020). Inferential analyses were performed using the *nlme* package (version 3.1-162) (Pinheiro, 2011) using two-tailed tests with a significance level of 0.05.

## Results

### Sample Characteristics and EMA Compliance

**Figure 1.**
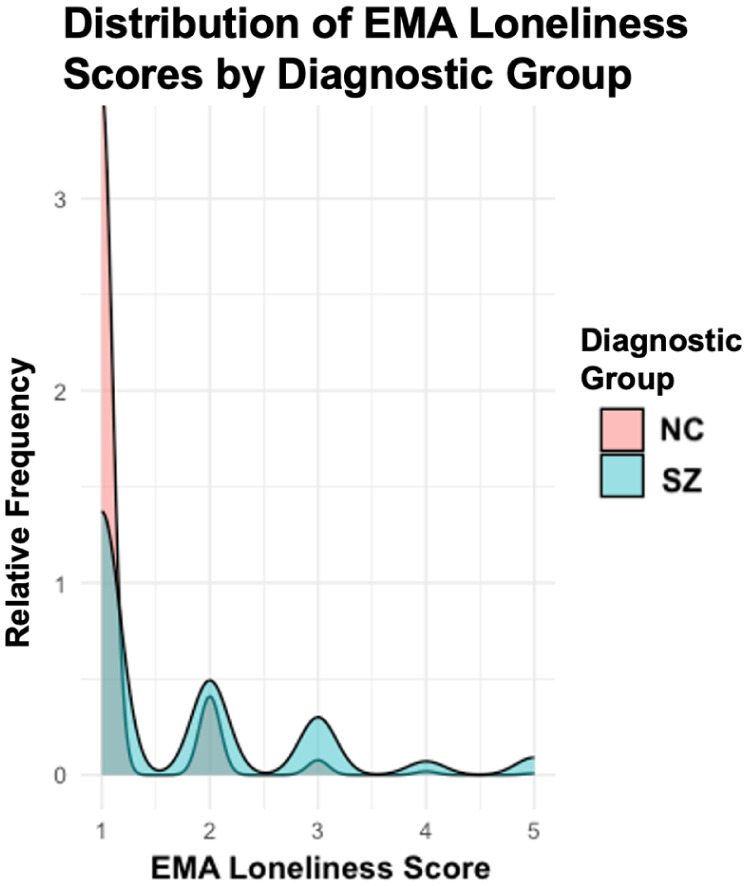
Distribution of EMA loneliness scores by diagnostic group.

Table 1 presents descriptive statistics for demographic characteristics, positive and negative psychosis symptoms, and loneliness measures by diagnostic group. People with schizophrenia reported significantly higher trait loneliness on the UCLA-LS compared to the NC group (*t* = 6.43, *p* < .001). Further, people with schizophrenia also reported higher intra-individual mean levels of state loneliness (see Figure 1). Within the schizophrenia group, mean scores on the SAPS and SANS suggest moderate levels of both positive and negative symptoms, respectively.

**Table 1.** Participant Characteristics and Loneliness Scores by Diagnostic Group.

	**SZ** (*n* = 39)	**NC** (*n* = 65)	*t* or		
Characteristic	M (*SD*) or **n (**%)	M (*SD*) or **n (**%**)**	$\chi^{2}$	df	*p*
Age (years)	53.8 (7.5)	57.3 (7.6)	−2.29	101	0.024
Gender			2.49		0.115
% Women	17 (43.6%)	40 (61.5%)		1	
Men	22 (56.4%)	25 (39.1%)			
Race/ethnicity			16.72	4	0.002
White	17 (43.6%)	47 (73.4%)			
Black	7 (17.9%)	2 (3.0%)			
Latine	9 (23.1%)	8 (12.3%)			
Asian	3 (7.7%)	8 (12.3%)			
Other Severity of psychopathology	3 (7.5%)				
Positive (SAPS total)	4.7 (3.6)	–			
Negative (SANS total)	5.2 (2.7)	–			
Average state loneliness (EMA)	1.7 (0.8)	1.2 (0.3)	−4.05	46.68	<.001
Trait loneliness (UCLA-LS total)	48.8 (9.6)	36.1 (9.7)	6.43	99	<0.001

*Note.* SZ = schizophrenia group; NC = Non-SMI Comparison group; SAPS = Scale for the Assessment of Positive Symptoms; SANS = Scale for the Assessment of Negative Symptoms; UCLA-LS = UCLA-Loneliness Scale–Third Edition; EMA = Ecological Momentary Assessment. Symptom ratings were not assessed in the NC group.

Given the schedule of four EMA surveys per day for 7 days, there were 28 potential EMA data points for each participant. 100% completion was defined as completing all 28 possible EMA loneliness questions on each EMA survey. Actual mean (and *SD*) completion rates were 84.0% (*SD* = 5.9%) for the NC group, and 77.3% (*SD* = 21.6%) for the schizophrenia group, which were not significantly different by the Wilcoxon Rank-Sum Test [*p* = 0.970].

### Symptom Severity and Loneliness Associations

Psychopathology ratings were not collected from participants in the NC group. Among participants with schizophrenia, greater trait loneliness was significantly associated with more severe positive symptoms (*r* = .39, *p* < .001) and more severe negative symptoms (*r* = .43, *p* < .001). Greater average state loneliness, based on participant-level means of EMA loneliness ratings, was also significantly associated with more negative symptoms (*r* = .25, *p* < .001), but was not significantly associated with positive symptom severity (*r* = .04, *p* = .26).

### Between- and Within-Person Variances of Loneliness EMA in People with Schizophrenia Versus Non-SMI Comparison Subjects

Relative to the NC group, people with schizophrenia showed significantly higher between-subject and within-subject variance in EMA reported loneliness ( $\sigma^{2}$ _*between*_ = 0.622 versus 0.111, *p* < .001; $\sigma^{2}$ _*within*_ = 0.548 versus 0.127, *p* < .001). Between-person variance reflects how much individuals differ from one another in their average levels of loneliness, while within-person variance reflects how much each individual fluctuates across time.

People with schizophrenia also had significantly higher intra-class correlation coefficient (ICC = 0.532) than the NC group (ICC = 0.465, *p* < .001), suggesting that a greater proportion of the total variance in loneliness was attributable to between-person differences. Of important note, the ICC is a proportional index of between-person to total variance and does not reflect the absolute size of within-person variability. In this case, people with schizophrenia showed higher variability in both dimensions.

**Figure 2.**
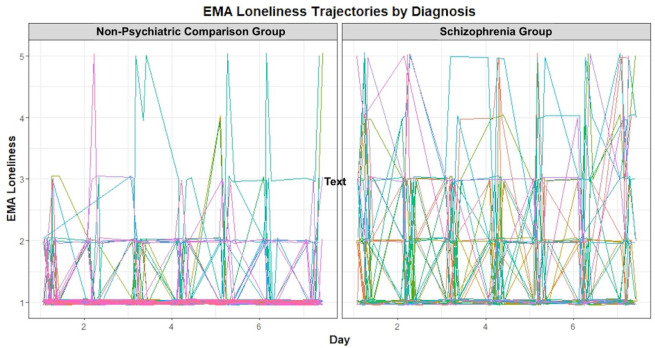
EMA loneliness trajectories by diagnostic group. *Note*. Each line represents participants’ EMA loneliness ratings across time.

When trait loneliness was included as a covariate, the pattern of results remained consistent. People with schizophrenia continued to show significantly higher between- and within-person variance than the NC group ( $\sigma^{2}$ _*between*_ = 0.508 versus 0.091, *p* < .001; $\sigma^{2}$ _*within*_ = 0.548 versus 0.127, *p* < .001), and ICCs remained significantly higher among people with schizophrenia (ICC = 0.481 versus 0.415; *p* < .001). From these results, we concluded that models that allowed for heterogeneous between-subject and within-subject variances were ideal for representing EMA loneliness data (see Figure 2).

### Loneliness Effect of Trait Loneliness Level on EMA Loneliness

Table 2 presents full parameter estimates and test statistics. Higher baseline UCLA-LS was significantly associated with higher EMA loneliness. Diagnostic group and gender were also significant in the model, with the schizophrenia group and men experiencing higher EMA loneliness overall. Covariates race/ethnicity and age were not statistically significant. The inclusion of UCLA-LS as a predictor resulted in lower estimates of between-subject variability without appreciably affecting within-subject variability estimates. The consequently lower ICC suggests that UCLA-LS helps explain a portion of between-subject variability in EMA loneliness.

**Table 2.** Fixed Effects from Linear Mixed-Effects Model Predicting EMA Loneliness.

Predictor	β	SE	*t*	df	*p*
Intercept	0.625	0.303	2.06	2364	.039
**Gender**	**0.161**	**0.079**	**2.05**	**95**	.**044**
**Diagnostic Group**	**0.283**	**0.142**	**1.99**	**95**	.**050**
**Trait Loneliness**	**0.015**	**0.003**	**4.65**	**95**	**<.001**
Age	−0.001	0.005	−0.19	95	.853
Race/Ethnicity					
Black	0.125	0.184	0.68	95	.500
Latine	0.061	0.113	0.54	95	.591
Asian	0.082	0.123	0.67	95	.508
Other	−0.267	0.445	−0.60	95	.550

*Note*. The reference group for the race/ethnicity variable is non-Latine White. Trait loneliness was measured by the UCLA-LS. Bolded coefficients and test statistics represent a two-tailed significance of *p* < 0.05.

## Discussion

Using EMA, the current study examined whether people with schizophrenia exhibit greater within-person variability in state loneliness compared to people without a history of serious mental illness (NC). Consistent with our hypothesis, people with schizophrenia had significantly higher variability in state loneliness compared to the NC group over 7 days of observation (including four daily surveys), totaling 28 maximum observations. Notably, this pattern remained consistent even after controlling for trait loneliness, suggesting that people with schizophrenia not only report higher levels of loneliness but also experience greater moment-to-moment fluctuations in how lonely they feel.

Our results extend a small but growing body of research using EMA to examine loneliness in schizophrenia (Culbreth et al., 2021; Moran et al., 2024; Mote & Fulford, 2020). However, prior work has less consistently examined within-person variability. The present study extends these findings by demonstrating that loneliness in schizophrenia is not only elevated at average levels but also more unstable across time, even after controlling for trait loneliness. This adds to emerging work suggesting that loneliness in schizophrenia may be more dynamic than previously appreciated when assessed using real-time methods.

Within the schizophrenia group, greater trait loneliness was moderately associated with both positive and negative symptoms, whereas state loneliness was significantly associated only with negative symptoms. Trait loneliness may tend to reflect broader psychological distress, including feelings of exclusion or being misunderstood, experiences that may overlap with positive symptoms such as suspiciousness or unusual beliefs (Badcock et al., 2015; Eglit et al., 2018). In contrast, state or momentary loneliness may be more sensitive to negative symptoms, such as reduced motivation or social withdrawal, that shape momentary feelings of disconnection (Culbreth et al., 2021; Moran et al., 2024). For instance, diminished anticipatory pleasure or avolition may reduce the drive to seek out and engage in social contact, leading to greater momentary awareness of being lonely. Conversely, positive symptoms such as paranoia or suspiciousness may reflect more persistent cognitive-affective disruptions that contribute to enduring feelings of loneliness, aligning more with trait loneliness. These distinctions could help clarify which symptom domains are most relevant when addressing chronic versus episodic loneliness in schizophrenia. While our findings should be interpreted with caution, given the small sample size, they underscore the value of modeling trait and state loneliness as distinct, yet clinically informative constructs.

Understanding what contributes to momentary fluctuations may offer insights into how loneliness manifests and intensifies in daily life among people with schizophrenia. The heightened within-person variability in state loneliness observed in our schizophrenia group may reflect increased emotional reactivity to daily social-contextual cues, consistent with prior EMA research showing that people with psychosis exhibit heightened sensitivity to minor daily stressors and affective triggers (Myin-Germeys & van Os, 2007). Momentary loneliness may co-occur with negative affect, which can be amplified by real-world stressors in this population. For example, a recent EMA study of individuals remitted from a first episode of psychosis found that negative affect (e.g., sadness, anxiety) but not psychotic symptoms or negative symptoms such as low motivation predicted momentary loneliness (Djordjevic et al., 2025). Our finding that momentary loneliness was not associated with positive symptoms aligns with evidence that, while momentary loneliness is linked to psychotic symptoms in high-risk samples, these associations might diminish after the first episode (Raposo de Almeida et al., 2024). This may be because positive symptoms tend to be episodic and less tightly tied to ongoing social behavior, particularly in later illness stages (Mucci et al., 2021; Tamminga et al., 1998). Together, these results highlight the importance of considering both illness stage and symptom domain when interpreting loneliness dynamics in schizophrenia. Finally, our findings add to emerging evidence that clinical assessments and EMA capture complementary facets of psychopathology in psychosis (Wright et al., 2021). Integrating both methods may offer a more comprehensive understanding of the stable and dynamic features of loneliness.

We also found that trait loneliness was significantly associated with average levels of state loneliness. However, trait loneliness did not account for the elevated within-person variability observed in people with schizophrenia, suggesting that it may explain between-person differences in mean loneliness but not the instability of these experiences over time. This supports growing recognition that trait and state loneliness, while related, reflect partially distinct processes (Maes & Vanhalst, 2024). Prior work has similarly shown that momentary loneliness can vary meaningfully beyond trait loneliness and may be shaped by contextual factors (Buecker et al., 2024; Mote & Fulford, 2020). Together with our findings, this highlights the importance of modeling both forms of loneliness in schizophrenia to improve assessment and intervention. Trait loneliness may reflect persistent social disconnection (Moran et al., 2024), while heightened within-person variability may point to reactivity in daily social experiences, both of which may warrant different clinical approaches.

### Limitations and Future Directions

One important caveat of the present findings is that the analyses were cross-sectional and correlational, limiting the ability to draw causal inferences about the relationship between trait loneliness and state loneliness. Second, state loneliness was assessed using a single EMA item, which, while commonly used in prior work, may not fully capture the nuance of momentary loneliness experiences. Loneliness is a multidimensional construct that can involve emotional, cognitive, and behavioral components, which may not be reflected in a single-item scale. Future work may benefit from incorporating multiple indicators or domains of momentary loneliness to better characterize its expression in real time. Third, our sample was restricted to middle-aged and older adults (ages 41–70), limiting generalizability to younger adults with schizophrenia. Loneliness may present differently earlier in the lifespan, especially given developmental differences in social context and motivation. Additional research is needed to determine whether age-related factors influence the magnitude or pattern of loneliness variability in schizophrenia. Fourth, given the limited sample size, we did not examine contextual predictors of momentary loneliness, such as the number of social interactions, location, or activity type. These variables may provide important insights into what drives daily shifts in loneliness. Moreover, we were not able to examine whether momentary increases in loneliness reflected adaptive processes (e.g., prompting reconnection) or maladaptive ones (e.g., leading to withdrawal or distress); a distinction may help to guide future intervention targets. Finally, because data collection relied on smartphone-based EMA, participant experience may have been affected by technological barriers, including variability in digital literacy.

Building on the distinction between trait and state loneliness, future research should explore how interventions might be tailored to these different loneliness profiles. While trait loneliness may require long-term strategies aimed at building enduring support structures, acutely heightened state loneliness may reflect a reactive or situational vulnerability that could benefit from just-in-time-adaptive interventions (JITAIs). These interventions use real-time data, such as EMA responses, to predict when and what type of support is most beneficial in the moment. JITAIs have demonstrated feasibility and acceptability in reducing loneliness among people with schizophrenia and other SMI (Gandhi et al., 2023; Hanssen et al., 2020; Lim et al., 2020).

The above caveats noted, the current findings still provide key new insights into the growing body of empirical literature on loneliness in schizophrenia. By using EMA and a within-person modeling framework, we were able to capture real-time fluctuations that revealed greater instability in loneliness among people with schizophrenia. Our findings build on longstanding theories that loneliness is not only a chronic condition, but also an episodic, affectively dynamic experience (Cacioppo & Cacioppo, 2018), particularly in the context of psychosis. Interventions targeting loneliness in schizophrenia may benefit from addressing both chronic and dynamic features of this experience, potentially improving well-being and social functioning in this highly marginalized population.

## References

[bibr1-00207640251383127] Andreasen N. C. (1984).Scale for the assessment of positive symptoms. Group, 17(2), 173–180.

[bibr2-00207640251383127] Andreasen N. C. (1989). The Scale for the Assessment of Negative Symptoms (SANS): Conceptual and theoretical foundations. The British Journal of Psychiatry, 155(S7), 49–52. 2695141

[bibr3-00207640251383127] Badcock J. C. Shah S. Mackinnon A. Stain H. J. Galletly C. Jablensky A. Morgan V. A. (2015). Loneliness in psychotic disorders and its association with cognitive function and symptom profile. Schizophrenia Research, 169(1–3), 268–273. 26527247 10.1016/j.schres.2015.10.027

[bibr4-00207640251383127] Buecker S. Horstmann K. T. Luhmann M. (2024). Lonely today, lonely tomorrow: Temporal dynamics of loneliness in everyday life and its associations with psychopathological symptoms. Social Psychological and Personality Science, 15(2), 170–181.

[bibr5-00207640251383127] Cacioppo J. T. Cacioppo S. (2018). Loneliness in the modern age: An evolutionary theory of loneliness (ETL). In Olson J. M. (Ed.), Advances in experimental social psychology (Vol. 58, pp. 127–197). Elsevier.

[bibr6-00207640251383127] Cavieres A. Acuña V. Arancibia M. Lopetegui N. (2023). Differences in social perception in people with schizophrenia and bipolar disorder. Schizophrenia Research: Cognition, 33, 100286. 37206445 10.1016/j.scog.2023.100286 PMC10189461

[bibr7-00207640251383127] Chen Y.-L. Jian C.-R. Chang Y.-P. Chao S.-R. Yen C.-F. (2023). Association of loneliness with suicide risk and depression in individuals with schizophrenia: Moderating effects of self-esteem and perceived support from families and friends. Schizophrenia, 9(1), 41. 37402821 10.1038/s41537-023-00368-7 PMC10319791

[bibr8-00207640251383127] Culbreth A. J. Barch D. M. Moran E. K. (2021). An ecological examination of loneliness and social functioning in people with schizophrenia. Journal of Abnormal Psychology, 130(8), 899. 34553952 10.1037/abn0000706 PMC9171710

[bibr9-00207640251383127] Dahlberg L. Agahi N. Lennartsson C. (2018). Lonelier than ever? Loneliness of older people over two decades. Archives of Gerontology and Geriatrics, 75, 96–103. 29220739 10.1016/j.archger.2017.11.004

[bibr10-00207640251383127] Djordjevic M. Jongsma H. E. Simons C. J. Oomen P. P. de Haan L. Boonstra N. Kikkert M. Koops S. Geraets C. N. Begemann M. J. , & others. (2025). Associations between momentary mental states and concurrent social functioning after remission from first episode psychosis: A HAMLETT ecological momentary assessment study. Journal of Psychiatric Research, 181, 560–569. 39708772 10.1016/j.jpsychires.2024.12.002

[bibr11-00207640251383127] Efron B. Tibshirani R. J. (1994). An introduction to the bootstrap. Chapman and Hall/CRC.

[bibr12-00207640251383127] Eglit G. M. Palmer B. W. Martin A. S. Tu X. Jeste D. V. (2018). Loneliness in schizophrenia: Construct clarification, measurement, and clinical relevance. PLoS One, 13(3), e0194021. 10.1371/journal.pone.0194021 PMC5863980 29566046

[bibr13-00207640251383127] Gandhi A. Mote J. Fulford D. (2023). The promise of digital health interventions for addressing loneliness in serious mental illness. Current Treatment Options in Psychiatry, 10(3), 167–180.

[bibr14-00207640251383127] Goldstein S. P. Dochat C. Schumacher L. M. Manasse S. M. Crosby R. D. Thomas J. G. Butryn M. L. Forman E. M. (2018). Using ecological momentary assessment to better understand dietary lapse types. Appetite, 129, 198–206. 29981361 10.1016/j.appet.2018.07.003 PMC13055932

[bibr15-00207640251383127] Hanssen E. Balvert S. Oorschot M. Borkelmans K. van Os J. Delespaul P. Fett A.-K. (2020). An ecological momentary intervention incorporating personalised feedback to improve symptoms and social functioning in schizophrenia spectrum disorders. Psychiatry Research, 284, 112695. 31831201 10.1016/j.psychres.2019.112695

[bibr16-00207640251383127] Hedeker D. Mermelstein R. J. (2007). Mixed-effects regression models with heterogeneous variance: Analyzing ecological momentary assessment (EMA) data of smoking. In Little T. D. Bovaird J. A. Card N. A. (Eds.), Modeling contextual effects in longitudinal studies (pp. 183–206). Lawrence Erlbaum Associates Publishers.

[bibr17-00207640251383127] Hussain M. A. Palmer B. W. (2024). Barriers to identifying and comparing rates of adaptive and maladaptive loneliness: Commentary for “Loneliness prevalence of community-dwelling older adults and the impact of the mode of measurement, data collection, and country: A systematic review and meta-analysis” by Stegen et al. International Psychogeriatrics, 36(9), 699–702. 39745344 10.1017/S104161022400067X

[bibr18-00207640251383127] Lim M. H. Gleeson J. F. Rodebaugh T. L. Eres R. Long K. M. Casey K. Abbott J.-A. M. Thomas N. Penn D. L. (2020). A pilot digital intervention targeting loneliness in young people with psychosis. Social Psychiatry and Psychiatric Epidemiology, 55, 877–889. 30874828 10.1007/s00127-019-01681-2

[bibr19-00207640251383127] Maes M. Vanhalst J. (2024). Loneliness as a double-edged sword: An adaptive function with maladaptive consequences. European Journal of Developmental Psychology, 22(4), 1–13.

[bibr20-00207640251383127] Manasse S. M. Crochiere R. J. Dallal D. H. Lieber E. W. Schumacher L. M. Crosby R. D. Butryn M. L. Forman E. M. (2018). A multimodal investigation of impulsivity as a moderator of the relation between momentary elevations in negative internal states and subsequent dietary lapses. Appetite, 127, 52–58. 29715502 10.1016/j.appet.2018.04.025 PMC10148240

[bibr21-00207640251383127] McComb S. E. Goldberg J. O. Flett G. L. Rose A. L. (2020). The double jeopardy of feeling lonely and unimportant: State and trait loneliness and feelings and fears of not mattering. Frontiers in Psychology, 11, 563420. 33391078 10.3389/fpsyg.2020.563420 PMC7773912

[bibr22-00207640251383127] Moran E. K. Shapiro M. Culbreth A. J. Nepal S. Ben-Zeev D. Campbell A. Barch D. M. (2024). Loneliness in the daily lives of people with mood and psychotic disorders. Schizophrenia Bulletin, 50(3), 557–566. 38429937 10.1093/schbul/sbae022 PMC11059807

[bibr23-00207640251383127] Mote J. Fulford D. (2020). Ecological momentary assessment of everyday social experiences of people with schizophrenia: A systematic review. Schizophrenia Research, 216, 56–68. 31874743 10.1016/j.schres.2019.10.021

[bibr24-00207640251383127] Mucci A. Galderisi S. Gibertoni D. Rossi A. Rocca P. Bertolino A. Aguglia E. Amore M. Bellomo A. Biondi M. , & others. (2021). Factors associated with real-life functioning in persons with schizophrenia in a 4-year follow-up study of the Italian network for research on psychoses. JAMA Psychiatry, 78(5), 550–559. 33566071 10.1001/jamapsychiatry.2020.4614 PMC7876615

[bibr25-00207640251383127] Myin-Germeys I. van Os J. (2007). Stress-reactivity in psychosis: Evidence for an affective pathway to psychosis. Clinical Psychology Review, 27(4), 409–424. 17222489 10.1016/j.cpr.2006.09.005

[bibr26-00207640251383127] Myin-Germeys I. van Os J. Schwartz J. E. Stone A. A. Delespaul P. A. (2001). Emotional reactivity to daily life stress in psychosis. Archives of General Psychiatry, 58(12), 1137–1144. 11735842 10.1001/archpsyc.58.12.1137

[bibr27-00207640251383127] Murthy V.H . (2023). Our epidemic of loneliness and isolation: The US Surgeon General’s Advisory on the healing effects of social connection and community. Office of the U.S. Surgeon General. https://www.ncbi.nlm.nih.gov/books/NBK595227/37792968

[bibr28-00207640251383127] Paolillo E. W. Bomyea J. Depp C. A. Henneghan A. M. Raj A. Moore R. C. (2024). Characterizing performance on a suite of English-language NeuroUX mobile cognitive tests in a US adult sample: Ecological momentary cognitive testing study. Journal of Medical Internet Research, 26, e51978. 10.2196/51978 PMC11629032 39586088

[bibr29-00207640251383127] Patterson T. Sajjadi F. Hobbs L. Barak Y. (2024). Loneliness in older persons with schizophrenia. International Journal of Social Psychiatry, 71(5), 844–852. 39713910 10.1177/00207640241307842 PMC12284327

[bibr30-00207640251383127] Pinheiro J. (2011). nlme: Linear and nonlinear mixed effects models. R Package Version, 3, 1.

[bibr31-00207640251383127] R Core Team. (2020). R: A language and environment for statistical computing, R Foundation for Statistical. Computing. [Computer software]. https://www.r-project.org/.

[bibr32-00207640251383127] Randolph S. B. Ratner A. M. Kersey J. Moran E. Barch D. M. Rousso B. Connor L. T. (2025). Exploring the experience of loneliness among people living with schizophrenia: A qualitative study. Issues in Mental Health Nursing, 46(1), 12–19. 39761214 10.1080/01612840.2024.2428632

[bibr33-00207640251383127] Raposo de Almeida E. van Der Tuin S. Muller M. K. van den Berg D. Wang Y.-P. Veling W. Booij S. H. Wigman J. T. (2024). The associations between daily reports of loneliness and psychotic experiences in the early risk stages for psychosis. Early Intervention in Psychiatry, 18(11), 930–942. 38661051 10.1111/eip.13537

[bibr34-00207640251383127] Russell D. W. (1996). UCLA loneliness scale (version 3): Reliability, validity, and factor structure. Journal of Personality Assessment, 66(1), 20–40. 8576833 10.1207/s15327752jpa6601_2

[bibr35-00207640251383127] Stain H. J. Galletly C. A. Clark S. Wilson J. Killen E. A. Anthes L. Campbell L. E. Hanlon M.-C. Harvey C. (2012). Understanding the social costs of psychosis: The experience of adults affected by psychosis identified within the second Australian National Survey of Psychosis. Australian & New Zealand Journal of Psychiatry, 46(9), 879–889. 22645395 10.1177/0004867412449060

[bibr36-00207640251383127] Tamminga C. A. Buchanan R. W. Gold J. M. (1998). The role of negative symptoms and cognitive dysfunction in schizophrenia outcome. International Clinical Psychopharmacology, 13, S21–S26. 10.1097/00004850-199803003-00004 9690966

[bibr37-00207640251383127] Taylor H. O. Nguyen A. Ojembe B. Tsuchiya K. (2025). Social isolation and loneliness among racial/ethnic minoritized older adults: A literature review. Clinics in Geriatric Medicine, 41(3), 449–461. 40653346 10.1016/j.cger.2025.04.009

[bibr38-00207640251383127] van’t Wout M. van Rijn S. Jellema T. Kahn R. S. Aleman A. (2009). Deficits in implicit attention to social signals in schizophrenia and high risk groups: Behavioural evidence from a new illusion. PLoS One, 4(5), e5581. 10.1371/journal.pone.0005581 PMC2680059 19440352

[bibr39-00207640251383127] Weittenhiller L. P. Mikhail M. E. Mote J. Campellone T. R. Kring A. M. (2021). What gets in the way of social engagement in schizophrenia? World Journal of Psychiatry, 11(1), 13. 33511043 10.5498/wjp.v11.i1.13 PMC7805250

[bibr40-00207640251383127] Wright A. C. Browne J. Skiest H. Bhiku K. Baker J. T. Cather C. (2021). The relationship between conventional clinical assessments and momentary assessments of symptoms and functioning in schizophrenia spectrum disorders: A systematic review. Schizophrenia Research, 232, 11–27. 34004382 10.1016/j.schres.2021.04.010

